# Is Chemoprophylaxis for Child Contacts of Drug-Resistant TB Patients Beneficial? A Systematic Review

**DOI:** 10.1155/2018/3905890

**Published:** 2018-04-02

**Authors:** C. Padmapriyadarsini, Mrinalini Das, Sharath Burugina Nagaraja, Mahalakshmi Rajendran, Richard Kirubakaran, Sarabjit Chadha, Prathap Tharyan

**Affiliations:** ^1^National Institute for Research and Tuberculosis, Chennai, India; ^2^Médecins Sans Frontières, New Delhi, India; ^3^ESIC Medical College and PGIMSR, Bangalore, India; ^4^Christian Medical College, Vellore, India; ^5^The International Union Against TB and Lung Disease, New Delhi, India

## Abstract

**Background:**

Preventive therapy for child contacts of multidrug-resistant tuberculosis (MDR-TB) patients is poorly studied, and no consensus about the role and the rationale of chemoprophylaxis has been reached.

**Objective:**

To conduct systematic review with an aim to determine the effectiveness of TB preventive therapy in reducing the incidence of TB disease in pediatric contacts of MDR-TB patients.

**Methods:**

We conducted a literature search for randomized control trials, cohort studies, and case reports of chemoprophylaxis for pediatric contacts of MDR-TB patients in PubMed, EMBASE, Cochrane Databases of Systematic Reviews, metaRegister of Controlled Trials, and other clinical registries through March 2017, using appropriate search strategy. In addition we searched abstracts from international conferences and references of published articles and reviews.

**Results:**

Of the 153 references assessed from various databases, seven studies were identified as relevant after adaption of eligibility criteria and assessed for systematic review. Of these, only two studies contributed data for the pooled meta-analysis.

**Conclusions:**

Though the available evidences suggest that the chemoprophylaxis for child contacts of MDR-TB patients is beneficial, data to support or reject preventive therapy is very limited. Further clinical research, in Tb endemic settings like India, needs to be performed to prove the beneficial effect of chemoprophylaxis for pediatric contacts of MDR-TB.

## 1. Introduction

The World Health Organization (WHO) estimates that, globally in 2016, there were around 600,000 incident cases of rifampicin-resistant tuberculosis (TB) of which 490,000 had multidrug-resistant tuberculosis (MDR-TB) with India, China, and Russian federation accounting for almost 47% of these cases [1]. The burden of childhood TB is estimated to be 10–15% of the total TB burden with an annual risk of TB infection at 2–5% [2]. About 5% of those infected are likely to develop the disease in the first year after acquiring the infection and another 5% during rest of their lifetime. In children MDR-TB is mainly due to primary transmission from the index case, rather than acquired from prior exposure to TB treatment. Thirty-five countries have reported at least one pediatric MDR-TB case between 1994 and 2011, with increased morbidity and mortality compared to drug-sensitive disease [3]. Early diagnosis and effective treatment of adult MDR-TB case remain the main strategy to reduce TB transmission. However, given the poor success rate of currently used treatment regimens, it is wiser to adapt other modalities to prevent the occurrence of MDR-TB among the countries who pledge to achieve End TB goal by 2035 [4]. One such strategy is to target the latent TB reservoir, with either drugs or vaccines, and prevent the progression of latent TB infection to active TB disease. But optimal chemoprophylaxis for pediatric contacts of MDR-TB patients is poorly studied, and no consensus about the role and the rationale of different preventive treatments has been reached. A systematic review in 2006 by Fraser et al. concluded that there is lack of studies from randomized controlled trials to confirm or refute the need for chemoprophylaxis for childhood contacts of DR-TB patients [5]. A recent systematic review by Marks et al. further added that though preventive therapy showed effectiveness in prevention of MDR-TB among contacts, they often resulted in treatment discontinuation due to the adverse effects from the drugs in the regimen [6].

Frequent monitoring of these children was emphasized as much as the need for more clinical trials to determine the drugs that will be effective for pediatric contacts of these patients. Therefore, to address the gaps in evidence and look for newer studies for chemoprophylaxis for child contacts of MDR-TB patients, a systematic review was carried out with the following objectives: (i) to determine the effectiveness of chemoprophylaxis or TB preventive therapy in reducing the incidence of tuberculosis disease in childhood contacts of drug-resistant pulmonary tuberculosis patients and (ii) to determine the occurrence of any adverse events during the course of chemoprophylaxis or TB preventive therapy.

## 2. Methods

A protocol for the systematic review was registered with PROSPERO and is available online. The search is up to date as on 31 June 2017: https://www.crd.york.ac.uk/PROSPERO/display_record.php?RecordID=39330.

### 2.1. Eligibility and Inclusion Criteria

#### 2.1.1. Types of Studies

We intended to include all randomized and quasi-randomized controlled trials (RCT): prospective and retrospective cohort studies and case reports of chemoprophylaxis for children of MDR-TB patients. Studies performed among children of MDR-TB patients without active disease, HIV infected or uninfected, and studies performed in any country and published in any language were included. Systematic reviews, policy papers/briefs, case reports, letters to the editors, programme reports, correspondence, and non-human studies were not included in the review.

#### 2.1.2. Types of Participants

All childhood contacts of MDR-TB patients between the age of 0 and 14 years were included. Definition of “contacts” was retained as they were defined in the original study. For example, Seddon et al. 2013 defined “contacts” as children < 5 years of age with significant exposure to an infectious (sputum smear of culture positive) pulmonary MDR-TB source patient [7].

#### 2.1.3. Types of Interventions

Interventions with chemoprophylaxis for MDR-TB of any duration were considered. Comparison in RCT was the control or comparator arm while the comparison in the cohort study was the standard of care.

#### 2.1.4. Types of Outcome Measures

The primary outcome measures were occurrence of TB disease or serious adverse events among childhood contacts of MDR-TB patients on chemoprophylaxis. The secondary outcomes were occurrence of adverse events and occurrence of MDR-TB among children on chemoprophylaxis.

#### 2.1.5. Search Strategy

We searched separately for randomized control trials, cohort studies, and case reports regardless of publication status (published and in press) in PubMed, EMBASE, Cochrane Databases of Systematic Reviews, and other clinical registries (WHO, United States, South Africa, and Australia-New Zealand clinical registry). The search was also extended to metaRegister of Controlled Trials (mRCT): ClinicalTrials.gov website to identify progressive trials and abstracts from international conferences: (i) The Union World Conference on Lung Health from 2000 to 2017 and (ii) the American Thoracic Society Conference from 2000 to 2017.

### 2.2. Data Collection and Analysis

#### 2.2.1. Selection of Studies

The systematic review was carried out in accordance with the Preferred Reporting Items for Systematic Reviews and Meta-Analyses guidelines. The search strategy was developed considering the population, intervention, comparator, and outcome of the systematic review. The following terms in PubMed were used for search: (((((((((Tuberculosis [MeSH Terms]) OR Latent tuberculosis [MeSH Terms]) OR Extensively Drug-Resistant Tuberculosis [MeSH Terms]) OR Tuberculosis, Multidrug-Resistant [MeSH Terms]) OR Tuberculosis, Pulmonary [MeSH Terms]) OR Mycobacterium tuberculosis [MeSH Terms])) AND ((((((Child [MeSH Terms]) OR child)) OR ((Pediatrics [MeSH Terms]) OR pediatric^*∗*^)) OR children)) AND ((“Family Characteristics”[Mesh]) OR contact tracing [MeSH Terms]))) AND (((chemotherapy) OR chemoprevention [MeSH Terms]) OR prophylaxis). Similar searches were carried out in all relevant electronic sources and stored as online bibliography in open access web-based application: Rayan (https://rayyan.qcri.org/). We also contacted the authors of ongoing clinical trials, journal editors of International Journal of TB and Lung Diseases, Public Health Action for manuscript in press, and experts in the field for details on the study.

The titles and abstracts of screened studies in bibliography were evaluated by PP, MD, and SBN independently for relevance according to the prespecified criteria.

#### 2.2.2. Data Extraction and Management

**Figure 1:**
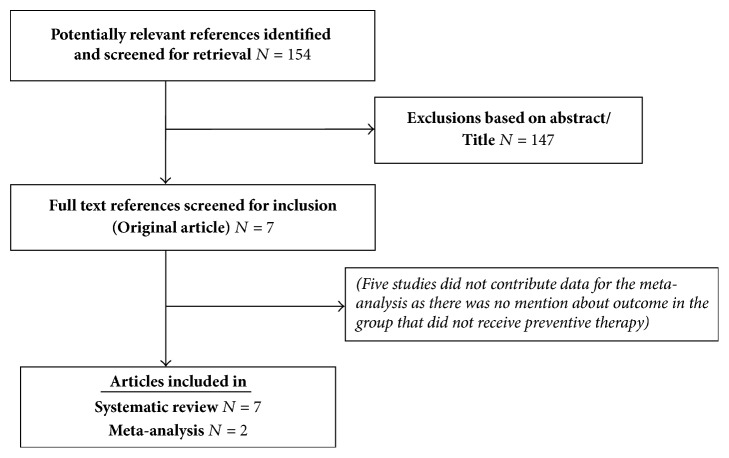
Search result of the topic.

Data were extracted individually by two authors (PP and MD) using standardized data extraction forms (Figure 1). Separate forms for randomized trials and prospective and retrospective cohort studies were used. We attempted to contact the trial authors for clarification when methodological details are unclear. Any disagreement was resolved by discussion with third author SBN.

#### 2.2.3. Data Synthesis

We assessed the similarity across the included studies on the characteristics of population intervention and the outcomes to provide a meaningful result to perform meta-analysis. Studies which did not describe the event or outcome in one of the groups—either the intervention group or the comparator group—were excluded from meta-analysis, though they will be included in the systematic review. We used Revman 5.3 software for meta-analysis. Mantel Hanzel method was used for dichotomous outcome reporting the relative risk along with 95% CI with fixed effect model. Inverse variance method was used for continuous data using fixed effect model presented with the mean difference with 95% CI [14]. We created subgroup according to type of intervention and age categories (0–5, 6–9, 10–14) and drug resistance profile and sensitivity analysis by excluding the high risk of bias studies. However, due to the limited data available, we did not undertake the subgroup and sensitivity analysis.

#### 2.2.4. Assessment of Heterogeneity

We assessed the heterogeneity with *i*2 statistics which describes the percentage of variation across the included studies rather than due to random error.

#### 2.2.5. Assessment of Risk of Bias

For randomized controlled trials Cochrane risk of bias tool was used for assessment following the standards given in the Cochrane handbook [15]. Downs and Black checklist was used for observational studies [16]. All the risk of bias figure was created in Revman 5.3 by modifying the domains depending upon the tool.

#### 2.2.6. Summary of Findings

Gradepro software was used for creating the summary of findings table. There are five domains for assessing certainty of evidence for randomized controlled trials (risk of bias, inconsistency, indirectness, imprecision, and publication bias). However for observational studies in addition to these existing domains the following domains were also considered (large effect, plausible confounding, and dosage response gradient).

Two authors (PP and MD) independently assessed the risk of bias of the selected studies using the Cochrane tool to assess the risk of bias and the quality of the evidence of the included studies was assessed using GRADE Pro software. Any disagreements were discussed with third author (SBN).

## 3. Findings and Results

The search strategy extracted 154 studies from the various databases published during January 2000 to June 2017; of these seven studies were considered potentially relevant (Figure 1). Three systematic reviews were identified and these were assessed for relevant references. Of these studies, after adaption of eligibility criteria, seven studies were included for the systematic review while only two studies were included for the pooled meta-analysis. 

### 3.1. Characteristics of Included Studies

The relevant characteristics of studies included are shown in Table 1. A study by Seddon et al. (2013) described the tolerability and toxicity of a standard MDR-TB preventive therapy regimen given to childhood contacts of adults with ofloxacin-susceptible MDR-TB and described the treatment outcomes [7]. This study included children below 5 years of age and children living with human immunodeficiency virus (HIV) infection below 15 years at the time of screening between May 2010 and April 2011. Children were initiated on preventive therapy with ofloxacin, ethambutol, and high dose isoniazid for a period of six months. A total of 186 childhood contacts were identified, given preventive therapy, and followed up for a period of at least 12 months.

Another study by Schaaf et al. (2002) described the long-term prevalence of tuberculous infection and disease in young children in household contact of adults with drug-resistant (DR) pulmonary TB in an area with high incidence of TB [8]. This study included 41 children below 5 years of age who were initiated on chemoprophylaxis according to the index's strain susceptibility and included high dose isoniazid, pyrazinamide, ethionamide, and ethambutol or high dose isoniazid, pyrazinamide, ethionamide, and ofloxacin daily for a period of six months. All children were followed up to thirty months.

Other studies on TB prevention for MDR-TB contacts included both adults and a small number of children and do not specify the outcome in children on preventive therapy or on placebo. For example, Adler-Shohet et al. (2014) described 31 children who developed latent infection after exposure to an index MDR-TB patient. Twenty-six were treated with levofloxacin and pyrazinamide; none developed TB but twelve required a change in therapy secondary to adverse effects [9]. However, there is no mention of any outcome in the five children who did not receive preventive therapy. Hence though this study was included for the systematic review, data from this study was not included for the meta-analysis. Similarly in the Garcia-Prats study, of the 38 who received preventive therapy, only 23 were children <5 years of age. Of them none developed TB. However among the comparator group (total = 4) who received no preventive therapy all seemed to be adults and no children [10]. Hence though these studies were included in the systematic review, they did not contribute data to the meta-analysis.

### 3.2. Effect of Intervention

**Figure 2:**
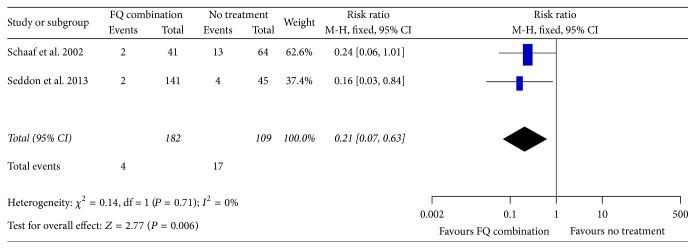
Forest plot of comparison of effect of preventive therapy versus no therapy for pediatric contacts of MDR-TB patients.

Between the two studies combined, there were four cases of TB in the contacts group that received chemoprophylaxis to prevent MDR-TB as compared to 17 cases of TB in the contacts group that did not receive any chemoprophylaxis (Figure 2). The relative risk for developing TB was 0.21 (95% CI 0.07–0.63). As the number of events were low, the confidence interval was wide. The other predefined outcomes were treatment completion rate, adverse events, and mortality rate. The pooled estimate of treatment completion rate was 0.74 (95% CI 0.64–0.82) among the included studies. Though adverse events were observed in all the studies, due to heterogeneity in the reporting of adverse events between the studies, a pooled estimate could not be calculated. There was only one reported death in the above-mentioned studies.

### 3.3. Risk of Bias Assessment

**Figure 3:**
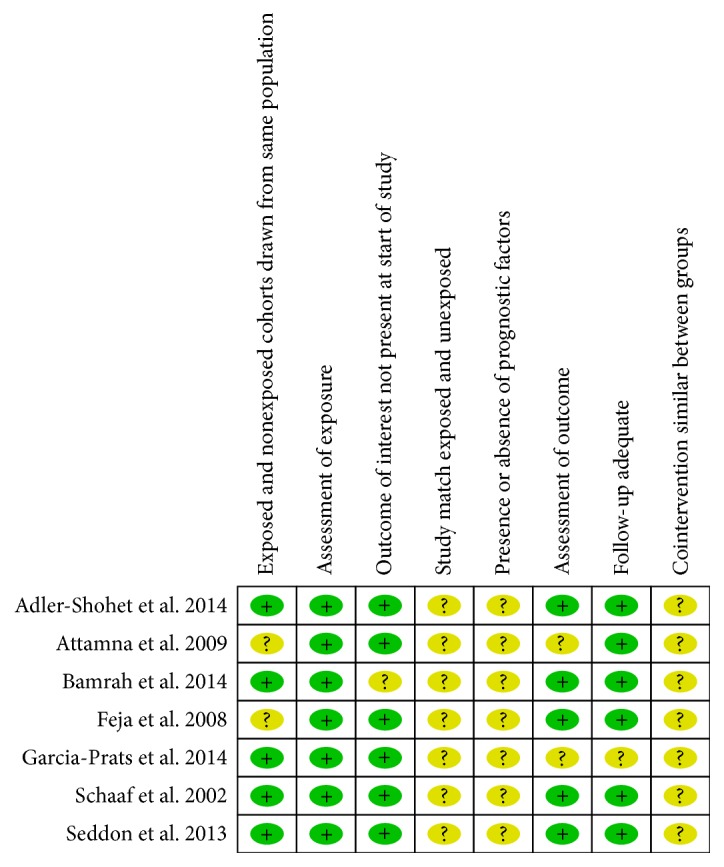
Summary of risk of bias assessment of the seven included studies.

Figure 3 shows the summary of the risk of bias assessment using the Black and Downs tool to assess the risk of bias for cohort studies. The tool assessed three domains, namely, (1) selection bias, (2) outcome ascertain bias, and (3) performance bias. A study could be awarded a maximum of “definitely yes,” showing a low risk of bias to a “definitely no” indicating high risk of bias for the three different domains. These studies do not mention any adjustments for confounders; hence, all have risk of bias.

### 3.4. Certainty of Evidence

The certainty of the evidence of the included studies was assessed using GRADE Pro software. As there were not any serious inconsistencies, imprecision, or indirectness in the results of the two studies, the quality of evidence could be upgraded. The quality of evidence for the effectiveness of chemoprophylaxis for child contacts of MDR-TB patients was thus assessed to be moderate (Table 2).

## 4. Discussions

This systematic review adds up to the evidences available on this topic in the scientific domain. Globally, the National Health Programmes have laid lot of emphasis on diagnosing and treating drug-resistant tuberculosis which is in line with the End TB strategy; however, the components of chemoprophylaxis for contacts of TB patients remain under prioritized or in primitive stage due to lack of standardized chemoprophylaxis regimens with respect to the drugs, dosages, and duration of therapy. The same has been opined in the recent systematic review by Fox et al. in 2017 [17].

We found that preventive therapy with second-line drugs, in line with the susceptibility profile of index case isolate or with fluoroquinolone, should be considered for chemoprophylaxis of contacts of MDR-TB patients. Our finding is supported by another retrospective review on pediatric contacts of MDR-TB patients in South Africa, where the authors concluded that children developed MDR-TB if they received chemoprophylaxis with either isoniazid or a combination of isoniazid, rifampin, and pyrazinamide and at least 2 second-line drugs should be considered for chemoprophylaxis of 6–12 months [18]. Other studies have reported varied results and the evidences are not sufficient to support or reject chemoprophylaxis for contacts of MDR-TB patients [19].

Currently, very few studies are conducted and published in this thematic area. Even the rigorous search in various databases of last two decades revealed only few studies. The quality of evidence provided by these studies is low to moderate and given the heterogeneity of these studies, one needs to be cautious while interpreting the results of these studies. However, there is evidence to support the reduction of risk of developing TB among contacts who receive chemoprophylaxis and the protection ranges from 37 to 90%.

All the studies reported adverse events irrespective of the type of drugs used. However, higher adverse events were recorded with ethionamide and pyrazinamide while fluoroquinolones had minimal toxicity. Discontinuation of treatment was noted among the contacts on ethionamide containing regimens predominantly due to gastrointestinal side effects.

To conclude, available evidences suggest that the chemoprophylaxis for child contacts of MDR-TB patient is beneficial, though the available evidence is of moderate quality. Further researches under controlled conditions in* TB endemic settings* are required to prove the beneficial or no effect of chemoprophylaxis for pediatric contacts of MDR-TB patients.

## Figures and Tables

**Table 1:** Summary of the seven studies showing their relevant details of included parameters.

Number	Author	Study design	Treatment regimen	Study subjects	Number of children	Treatment completion rate	Adverse event	Incidence TB	Mortality	Follow-up months	Remark
(1)	Seddon et al. [7] (2013)	Prospective cohort South Africa	Ofloxacin, ethambutol, and high dose isoniazid^*∗*^	Children < 5 years, CLHIV < 15 years	186	75.8% (141/186)	7/186 (3.7%)	6/141 on PT 4/45 not on PT	1 (0.5%)	219 p-y (12–24 months)	-
(2)	Schaaf et al. [8] (2002)	Prospective cohort S. Africa	High dose isoniazid, pyrazinamide, ethionamide, ethambutol/ofloxacin^*∗*^	Children < 5 years old	41 + 64		30/61 Eth 1/15 OFX	2/41 on PT 13/64 not on PT	No deaths	30	-
(3)	Adler-Shohet et al. [9] (2014)	Prospective cohort California	9 mon levofloxacin and pyrazinamide	Children	31	8/26 (31%) (7 changed Rx 11 did not complete)	All children	0	No deaths	24	*No separate mention on outcome in those who refused PT*
(4)	Gracia-Prats et al. [10] (2014)	Retrospective community cohort S. Africa	Ofloxacin, ethambutol, and high dose isoniazid^*∗*^	<15 years	38	24/34 (71%)	No	No TB	No deaths	12	*PT offered only to HHC < 5 years of age*
(5)	Bamrah et al. [11] (2014)	Prospective cohort Micronesia	12 mon levofloxacin and ethambutol	Children < 12 years	119 *(only 26 children)*	25/26 (95%)	Not clear	0/104 on PT 3/15 not on PT	No deaths	36	*Not mentioned who developed TB—adult or child*
(6)	Feja et al. [12] (2008)	Retrospective chart review NYC	9 mon quinolones, cycloserine, ethionamide, pyrazinamide, ethambutol	Children < 15 years	51	36/41 (88%) at DOHMH; 2/9 (22%)	8/22 (24%)	No TB	No deaths	24	*No comparator group without PT *
(7)	Attamna et al. [13] (2009)	Retrospective chart review Israel	Ciprofloxacin and pyrazinamide^*∗*^		476 (387 no Rx versus 89 PT)	Not clear	Not clear	No TB in any group	Not clear	72	*Low TB prevalence area*

**Table 2:** Quality of evidence and results from included studies.

Quality assessment							Number of patients		Effect		Quality	Importance
Number of studies	Study design	Risk of bias	Inconsistency	Indirectness	Imprecision	Other considerations	Preventive therapy	No preventive therapy	Relative (95% CI)	Absolute (95% CI)		
Incidence of TB												
2	Observational studies	Not serious	Not serious	Not serious	Not serious	Strong association	4/182 (2.2%)	17/109 (15.6%)	RR 0.21 (0.07 to 0.63)	123 fewer per 1,000 (from 58 fewer to 145 fewer)	*⨁⨁⨁*◯ Moderate	
